# Understanding epidemiological transition in India

**DOI:** 10.3402/gha.v7.23248

**Published:** 2014-05-15

**Authors:** Suryakant Yadav, Perianayagam Arokiasamy

**Affiliations:** International Institute for Population Sciences, Mumbai, India

**Keywords:** noncommunicable diseases, communicable diseases, disease patterns, mortality transition, epidemiological transition

## Abstract

**Background:**

Omran's theory explains changing disease patterns over time predominantly from infectious to chronic noncommunicable diseases (NCDs). India's epidemiological transition is characterized by dual burden of diseases. Kumar addressed low mortality and high morbidity in Kerala, which seems also to be true for India as a country in the current demographic scenario.

**Methods:**

NSS data (1986–1987, 1995–1996, 2004) and aggregated data on causes of death provided by Registrar General India (RGI) were used to examine the structural changes in morbidity and causes of death. A zero-inflated poisson (ZIP) regression model and a beta-binomial model were used to corroborate the mounting age pattern of morbidity. Measures, namely the 25th and 75th percentiles of age-at-death and modal age-at-death, were used to examine the advances in mortality transition.

**Objective:**

This study addressed the advances in epidemiological transition via exploring the structural changes in pattern of diseases and progress in mortality transition.

**Results:**

The burden of NCDs has been increasing in old age without replacing the burden of communicable diseases. The manifold rise of chronic diseases in recent decades justifies the death toll and is responsible for transformation in the age pattern of morbidity. Over time, deaths have been concentrated near the modal age-at-death. Modal age-at-death increased linearly by 5 years for females (*r*^2^=0.9515) and males (*r*^2^=0.9020). Significant increase in modal age-at-death ascertained the dominance of old age mortality over the childhood/adult age mortality.

**Conclusions:**

India experiences a dual burden of diseases associated with a remarkable transformation in the age pattern of morbidity and mortality, contemporaneous with structural changes in disease patterns. Continued progress in the pattern of diseases and mortality transition, accompanied by a linear rise in *e*_*x*_, unravels a compelling variation in advances found so far in epidemiological transition witnessed by the developed nations, with similar matrices for India.

The parallel processes of demographic and epidemiological transition are currently occurring at remarkable speed in India. From a comparative perspective, in the absence of sufficient longitudinal data from the vital registration system, the study of epidemiological transition has received much less attention to adequately understand the major shifts in mortality and morbidity patterns (1–3). Since the 1970s, researchers have primarily focused on India's demographic transition due to the availability of good long-term demographic trend data on mortality, fertility, and population growth from a sample registration system (SRS) (4–9). Aside from this, the limited number of studies that have addressed the broader domain of India's health transition (comprising mortality and epidemiological transition) have been narrowly focused because of data constraints. In general, the historical trends in mortality conditions of low- and middle-income countries have varied from those of high-income countries. For example, in underdeveloped nations such as India, mortality began to fall (10, 11) around the 1950s but accelerated through the 1970s and 1980s. For example, although the infant mortality rate (IMR) fell by two-thirds, from 129 to 44/1,000 live births, the crude death rate was reduced by half, from 14.9 to 7.1/1,000 persons, during the period 1970–2011 (12, 13). In an era of such emerging trends, India and its states have transitioned from high mortality conditions to medium/low mortality conditions in the last three decades.

Accumulated data from multiple sources suggest that, consistent with the current phase of demographic and epidemiological transition, the pace of ‘India's Health Transition’ has been swift (14, 15). Furthermore, the progress in health transition has spread over all geographical regions of India where the rise in morbidity is accompanied by a marked fall in mortality. Nevertheless, data also suggest notable heterogeneity among states with the highest morbidity rates (such as 255/1,000 persons in Kerala) and the lowest (in poorer states such as Jharkhand where the rate is 33/1,000 persons). Further data from India’s National Sample Survey Organization (NSSO) revealed an enormous increase in India's morbidity level during the last two decades (16–18). The rise of noncommunicable diseases (NCDs) has been associated with risk factors such as low physical activity, use of tobacco, low intake of fruits and vegetables, high body mass index (BMI), and so on. However, the government of India (GOI) has recognized that, irrespective of these risk factors and of socioeconomic status, NCDs are extremely common among older people. Between 1995–1996 and 2004, the crude morbidity rate increased by more than 60 and 90%, respectively, among rural and urban populations. In both rural and urban areas, the rise in morbidity level has been common across demographic and social spectrum:—among females, males, social groups, monthly per capita expenditure (MPCE) classes, and so on (16, 19).

In order to understand India's progress in epidemiological transition, in this paper we assess that country's structural changes in patterns of morbidity and mortality. Omran's (20, 21) theory of epidemiological transition provides a useful basis for examining such shifts in mortality and morbidity. The theory describes the process of changing disease patterns over time—predominantly from infectious to chronic NCDs (20, 21). However, the theory has been benchmarked primarily based on the experiences of developed nations where the burden of chronic NCDs has replaced the burden of communicable diseases. Contrastingly, in developing countries such as India, communicable diseases have been an additional burden due to the mounting number of NCDs (15, 16, 22). As a result of such confounding patterns (with e_0_ equaling 66.1 years in 2006–2010), epidemiological transition in India may be moving through the *Age of Receding Pandemics* and the *Age of Degenerative and Man-Made Diseases* (23). Progression to a particular stage of epidemiological transition is important to investigate while India is in the midst of this swift transition.

Overall, India has been experiencing rapid structural changes in disease patterns within the short span of the last three decades. Advances in mortality and morbidity transition to later stages point toward an upheaval in epidemiological transition. Visaria (15) has brought to light the phenomenon of the dual burden of disease and its linkages with epidemiological transition. In rural India, the burden of NCDs increased from 35.9 to 54.9%, and the burden of communicable diseases declined from 47.7 to 22.1%, during the transitional period from the 1970s to the mid-1990s (15). Although the overall burden of communicable diseases has declined, this burden is still very significant. More importantly, the share of communicable diseases has not been entirely replaced by chronic NCDs. In addition to Visaria's study (15), John et al. (24) have recently demonstrated the heavier burden of communicable diseases, which is as high as 30% of the total. Therefore, India's current stage of epidemiological transition can be characterized by low mortality, high morbidity, and by the double burden of communicable diseases and NCDs. These broad mortality and morbidity trends suggest that India represents a major contrast in the process of epidemiological transition particularly in light of conclusions based on studies of developed nations.

Viewed from an analytical context, the structural changes in disease patterns are concomitant with the transformation in the age pattern of morbidity and mortality. The age pattern of mortality (25) has been flattening over decades; however, the age pattern of morbidity is not commensurate with the transformation in age pattern of mortality. Rather, in recent decades, the age pattern of morbidity has been mounting as a result of the increasing prevalence of NCDs and communicable diseases (19, 26, 27). Altogether, these intervening components stimulate the progression of mortality transition in the country (28) thereby increasing the progress of epidemiological transition. This fundamental structural shift possibly signals advances in epidemiological transition (26, 29, 30). Researchers have acknowledged this phenomenon and have studied this variation in epidemiological transition in India (an underdeveloped nation) compared to the progress of epidemiological transition so far observed in developed nations. Regardless, rarely any of the studies have addressed the India's variation in its progress of epidemiological transition from the globally established course of epidemiological transition. Therefore, it is critical to fill this theoretical research gap for underdeveloped nations. This paper attempts to accomplish this by investigating fundamental processes such as the age pattern of morbidity, structural changes in disease patterns, and mortality transition to assess the course of India's epidemiological transition. The specific objectives explored are: 1) the prevalence of chronic diseases among the population aged 60 and above, 2) structural changes in causes of death, and 3) transformation in distribution of age-at-death and modal age-at-death. The first two objectives explain the correlation between morbidity and causes of death, and the third objective connects it with mortality transition. Broadly, the study addresses whether the structural changes in disease patterns conform to the progression in mortality transition and hence to the advances in epidemiological transition.

## Background

The burden of communicable diseases had been dominant around the world until the mid-twentieth century. In Africa and Asia (including India), the trilogy of smallpox, plague, and malaria remained the ‘top killer’ diseases until the 1960s. However, by the late 1970s, smallpox was eradicated in India through sustained vaccination programs; better sanitation and housing reduced the overall burden of communicable diseases (31). Nevertheless, by the mid-1990s, other communicable diseases, such as tuberculosis of lungs (5.3%), gastroenteritis/dysentery (3%), and pneumonia (4.7%) were responsible for a considerable number of deaths, signifying the burden of communicable diseases. Overall, the manifold rise in morbidity rates in recent decades concomitant with a decline in mortality rates is a manifestation of the progression of ‘health transition’ (32); this is a phenomenon where the survival rate of the population led to an increase in the level of morbidity and a decline in mortality. Kumar (14) explored the rapidly advancing phenomenon of ‘health transition’ in Kerala because this state had been experiencing consistently high morbidity prevalence levels with the steep fall in mortality (14). The phenomenon expedites the process of expansion of morbidity, which is found to be true at the national level by residence and by sex (26, 33).

The advances in mortality transition were explored by Ranjan Chaurasia (28) for the period 1970–2005 based on a measure of ‘entropy’ in life expectancy tables. He found the level of mortality continues to be high based on international standards. Among females, the pace of mortality transition slowed down. Comparatively, among urban males, mortality transition was found to be comparatively faster.

Today, modal age-at-death is widely used for testing mortality transition. Canudas-Romo (34), while testing the shifting mortality hypothesis, studied the modal age-at-death using various models, such as the Gompertz, Logistic, Siler, and Log-Siler models. The Siler model revealed a rise in modal age-at-death alongside a shift in distribution of age-at-death, although the Gompertz model only showed a shift in the distribution of age-at-death. The earlier models assume a reduction in IMR over time, while the later model assumes IMR is at the lowest possible value. The results were affirmative; modal age-at-death addressed the change in mortality. Nevertheless, a declining IMR and adult mortality rate have an impact on the modal age-at-death and on subsequent transformation in distribution of age-at-death. Modal age-at-death, similar to entropy and life expectancy, differentiates the pace of mortality transition among different categories of population. Additionally, modal age-at-death is a measure of length of life and is relevant for measuring dispersion of distribution of age-at-death (35). Modal age-at-death is useful for understanding changes in life expectancy and corresponding changes in the age pattern of mortality. The reduction in dispersion above the modal-age-at-death accompanied by the concentration of death in a narrower age-interval indicates a high to low mortality transition. Trends in C50—a measure of mortality compression—and modal age-at-death demonstrated virtual convergence among the developed countries in the last stage of epidemiological transition (35–37). Modal age-at-death is sensitive, simple, and convenient to use and has multiple applications, and therefore is preferred over other measures. Accordingly, it has been used globally to assess phenomena such as mortality transition and demographic and epidemiological transition. It is worthwhile to mention that the transformation in distribution of age-at-death is a simpler way to address phenomena such as mortality compression, inequality in age-at-death, and mortality transition (38).

## Data and methods

In this study, we have used multiple data sources and integrated them together for national level analysis. First unit level cross-sectional NSSO data for 1986–1987, 1995–1996, and 2004 on morbidity of aged (60+) persons were used to understand the changes in the prevalence rate of chronic diseases over time. The NSSO has retained the specific section on morbidity and ailing persons for the aged population for the three time periods (1986–1987, 1995–1996, and 2004); however, data on morbidity/NCDs for all ages is available only for the time periods 1995–1996 and 2004. The coverage of chronic diseases specific to the aged population remained same for the three time periods. Also, the prevalence of chronic diseases has escalated remarkably among the aged population signaling rapid changes in the morbidity profile of India (17, 18, 26). Therefore, morbidity analyses were done for the population aged 60 and above for the three data points. The prevalence rate of chronic diseases—hypertension, joint and bones, asthma, heart disease, cancer and other tumors, urinary problems, and diabetes—was adjusted for age, sex, residence, living alone, dependency, hospitalization, education, MPCE, and region (north, east, northeast, west, south, and central) using a ZIP regression model. We used a beta-binomial model to examine the changes in summary event rate of chronic diseases and also in total NCDs over time for the older population. The occurrence of chronic diseases/NCDs in a household of an older person age 60 or above with a reference period of the last 15 days will be a success, otherwise failure of the event. Thus, the probability of the occurrence of chronic diseases/NCDs for an older person in a household follows binomial distribution. However, the probability of occurrence of chronic diseases/NCDs across households varies and follows a beta distribution. Therefore, the application of a compound distribution —beta-binomial distribution—allows estimation of occurrence probability for the summary event rate of chronic diseases/NCDs for the older population across households (39). NSS 1986–1987, 1995–1996, and 2004 provide information for 8,478; 7,016; and 18,261 households, respectively, for ailing older persons aged 60 and above.

Second, we used aggregated data by broad age groups from a survey of causes of death (SCD) for rural areas (40) and mortality statistics of causes of death (MSCD) for urban areas (41) to understand the structural changes in causes of death. Medical certification of causes of death (MCCD) provided mortality statistics mostly for urban areas (42). For the period 2001–2003, RGI provided mortality statistics for both rural and urban populations (22). These data have been used together to construct the distribution of deaths attributable to communicable diseases and to NCDs for rural and urban India, respectively, and to examine the transformation in distribution of deaths.

Third, an age specific death rate (ASDR) provided by RGI (25) was used to construct new life tables based on the methodology proposed by the United Nations (43–45). The _5_a_x_ values of the life tables were very close to 2.5, which confirms the uniform distribution of age-at-death in the age groups. Therefore, the *d*_*x*_ column of the life table, for the age group 10 and above (46), was disaggregated into single years using the King-Karup method (47). Furthermore, the single-year distribution of age-at-death was smoothed using the cubic spline method to remove the erratic fluctuations (48, 49). Henceforth, the truncated and smoothed distribution is referred to as distribution of age-at-death. The transformation in distribution of age-at-death, trends in the 25th and 75th percentiles of age-at-death and at modal age-at-death are mainly examined to understand the progress of mortality transition in India (34, 35). The 25th and 75th percentiles of age-at-death refer to the age corresponding to 25 and 75% of deaths in distribution of age-at-death; modal age-at-death refers to the age corresponding to the modal value of the distribution of age-at-death.

## Results

In this section, we present the results of our analysis on 1) structural changes in morbidity and causes of death and 2) transformation in distribution of age-at-death and modal age-at-death. The results overall describe the linkage between morbidity pattern and causes of death and the consequent progress in epidemiological transition.

### Structural changes in morbidity

**Fig. 1:**
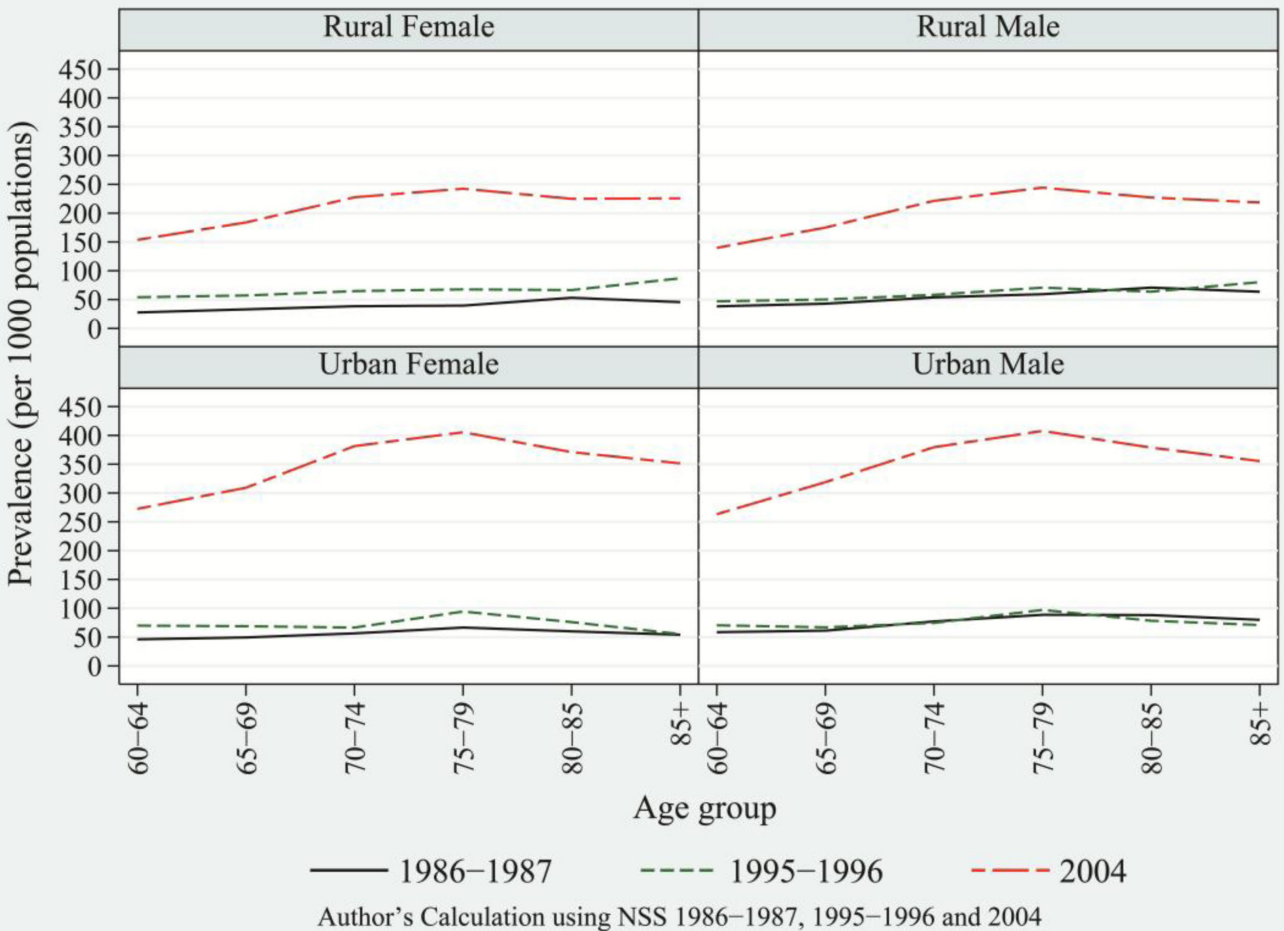
Age pattern of prevalence rate of chronic diseases by sex and residence, India for 1986–1987, 1995–1996, and 2004.

Figure 1 displays the age pattern of chronic diseases—by residence and by sex—for 1986–1987, 1995–1996, and 2004. The age pattern of morbidity reveals a mounting concentration of morbidity prevalence in the 60–64 and older age groups. The rising gradient of morbidity prevalence in the older ages peaked at the old–old (70–79) ages of 75–79. As a result of such a sudden and steep rise in the prevalence of chronic diseases, the overall morbidity began to take a distinguishing shape in 1995–1996 compared to the pattern observed among the developed nations. The prevalence rate of chronic diseases for ages 60+ increased from 48.5 in 1986–1987 to 69.5 in 1995–1996 and rose to 260.8 in 2004; overall, this indicated a more than five-fold rise in the prevalence of chronic diseases. Table 1 provides trends in the prevalence rate of each chronic disease among the aged population by sex and residence. By sex and residence, the prevalence rate of chronic diseases was highest among urban males and was characterized by a high prevalence among old–old and oldest of old (ages 80 and above). Among young–old (ages 60–69), the prevalence rate was relatively low compared to old–old and oldest of old. The prevalence rate for cardiovascular diseases (CVDs), which includes heart disease and hypertension, increased from 14.9 in 1986–1987 to 19.5 in 1995–1996 and escalated to as high as 63 in 2004, representing four-fold rise during the period of 1986–1987 to 2004.

**Table 1:** Prevalence rate of chronic diseases among older population by residence and sex, India, 1986–1987, 1995–1996, and 2004

	1986–1987				1995–1996				2004			
			
	Rural		Urban		Rural		Urban		Rural		Urban	
						
Chronic diseases	F	M	F	M	F	M	F	M	F	M	F	M
Asthma/cough and acute bronchitis*	10.3	17.9	11.2	19.4	14.5	18.4	8.4	12.2	19.2	33.7	18.8	30.6
Problems of joints and bones	10.7	11.2	9.0	8.3	27.6	18.0	22.3	14.0	52.9	33.3	61.1	41.1
Hypertension	7.1	6.7	19.8	18.8	11.1	6.5	20.4	16.1	28.6	21.4	80.0	60.2
Heart disease	2.1	3.3	3.8	6.3	3.4	5.7	7.0	15.4	10.0	14.0	31.7	45.3
Urinary problems	0.5	1.3	0.8	2.1	1.7	2.6	2.2	3.7	3.3	6.8	3.1	7.3
Diabetes	1.3	2.2	4.3	7.3	3.7	4.7	12.1	15.5	14.3	15.4	49.6	57.3
Cancer and other tumors**	0.4	0.5	0.6	0.7	1.5	1.8	1.7	1.8	3.6	2.4	4.6	3.2

Source: Author's calculation from NSS 1986–1987, 1995–1996, and 2004; F: female; M: male.

* In 1995–1996, information was provided for cough and acute bronchitis. In 1986–1987 and 2004, information was provided for asthma.

** In 1986–1987, information was provided for cancer only. In 1995–1996 and 2004, information was provided for cancer and other tumors.

In general, the age pattern of communicable diseases does not correspond with the changing age pattern of chronic disease. This is because the change in the prevalence rate of communicable diseases has not been sufficient to mark any structural changes in the overall age pattern of morbidity (26). In contrast, the enormous rise of degenerative diseases compared to any other categories of diseases, and the increased fatality rates due to them, have been largely responsible for deaths in older ages and, consequently, have contributed to the structural changes in disease patterns. The overall rise in degenerative diseases in recent decades indicates progress in the epidemiological transition to a later stage.

It is important to validate the enormous rise of chronic diseases because a remarkable increase in chronic NCDs has been revealed. The beta-binomial model was tested to estimate the summary event rate of chronic diseases, which takes into account the variation in the chance of occurrence of chronic NCDs across households and the binomial distribution followed by chronic diseases in each household (Table 2) (39). The summary event rate of chronic diseases increased from 4.01% (0.08%) in 1986–87 to 4.6% (0.22%), and to 12.7% (0.23%) in 2004. The trends in summary event rate of chronic diseases reveal a steep rise between 1995–1996 and 2004 compared with the marginal increase between 1986–1987 and 1995–1996. The parameters of the beta-binomial model give alpha and beta estimates of 0.05 and 1.18, respectively, for 1995–1996 and 0.16 and 1.1, respectively, for 2004. The likelihood ratio test for overdispersion was significant at the 1% level of significance for both years and thus confirmed the applicability of the beta-binomial model over the binomial model. The test was done for NCDs for 1995–1996 and for 2004 (3). The summary event rate of chronic NCDs rose by more than three times between 1995–1996 and 2004, similar to the manifold rise of chronic diseases.

**Table 2:** Summary event rate for chronic diseases, India, 1986–1987, 1995–1996, and 2004

Year	Event rate (SE) %		Dispersion (SE) %		α	β	2 (LBB-LB)
1986–1987	4.01	0.08	60.70	4.50	0.06	1.57	870.85***
1995–1996	4.60	0.22	80.30	9.20	0.05	1.18	440.65***
2004	12.70	0.23	79.10	5.30	0.16	1.10	930.42***

Source: Author's calculation from NSS 1986–1987, 1995–1996, and 2004. LBB: Log Likelihood of Beta Binomial model; LB: Log Likelihood of Binomial model.

*** Significant @ 1% level of significance.

The parameters of the beta-binomial model and the test of overdispersion are given in Table 3. Between 1995–1996 and 2004, chronic diseases and NCDs show a similar manifold rise that corroborates the rises in the prevalence rate obtained from the regression model. In sum, the several-fold rises of chronic diseases certainly transformed the age pattern of morbidity. This transformation in the age pattern of morbidity has been rapid since the mid-1990s. In recent decades, degenerative diseases have been responsible for poor health in old ages with an increase in life expectancy in old ages (26). Accordingly, they have been accountable for the heavy burden of deaths in old ages. This phenomenon is indicative of the increasing share of deaths due to NCDs and of the corresponding advances in epidemiological transition.

**Table 3:** Summary event rate for noncommunicable disease, India, 1995–96 and 2004

Year	Event rate (SE) %		Dispersion (SE) %		α	β	2 (LBB-LB)
1995–1996	5.10	0.22	77.20	8.60	0.06	1.23	439.08***
2004	16.00	0.25	79.20	4.90	0.20	1.06	930.42***

Source: Author's calculation from NSS 1995–1996 and 2004.

*** Significant @ 1% level of significance.

### Structural changes in causes of death

Structural changes in overall disease patterns over a period of 30 years and more, with the concomitant transformation in the age pattern of morbidity and mortality, resulted in significant structural changes in causes of death (50–53). During the 1970s and 1980s, infectious and parasitic diseases were the dominant cause of death in India. Among rural populations, diarrhea (diseases of the digestive system), cough (disorders of the respiratory system), and fever were responsible for 8, 23 and 15.5%, respectively, of the total deaths in 1972. The share of these diseases declined to 36 and 31% (40), respectively, in 1982 and 1997 with a considerable share of deaths attributable to tuberculosis of the lungs, gastroenteritis/dysentery, and pneumonia. Among the urban population, infectious and parasitic diseases, diseases of the respiratory system, and diseases of the digestive system, were responsible for 26, 10 and 6%, respectively, of total deaths in 1975. In 1985, these diseases accounted for 36.4% of total deaths, but this figure declined to 28.1% in 1995 (41). Among the urban population, the burden of respiratory tuberculosis and pneumonia accounted for an average of 5 and 3%, respectively, of total deaths from 2001 to 2004 (42).

**Fig. 2:**
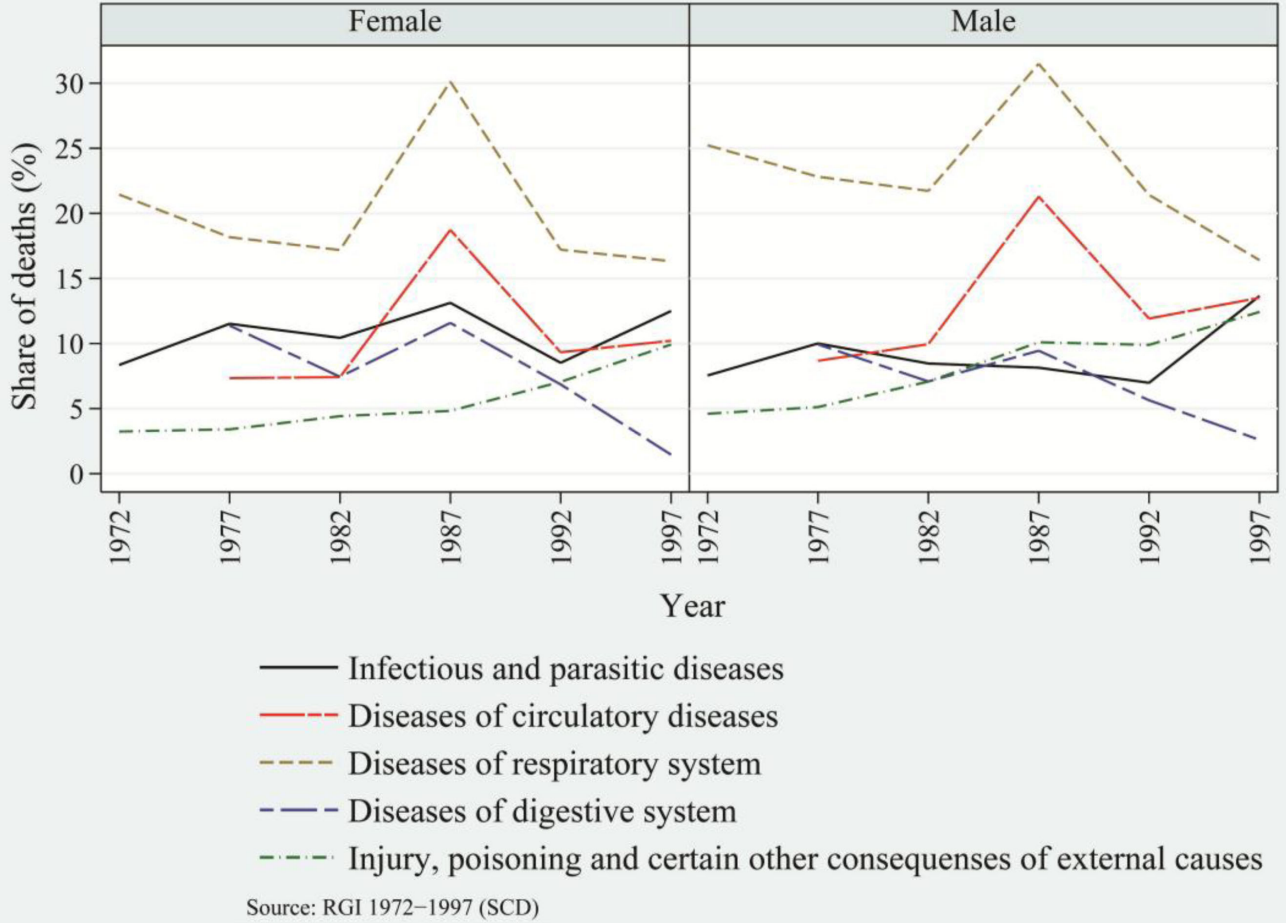
Share of major causes of death among females and males, rural India, 1975–1995: Infectious and parasitic diseases, circulatory diseases, respiratory diseases, diseases of the digestive system, injury, poisoning, and certain other external causes, such as senility.

**Fig. 3:**
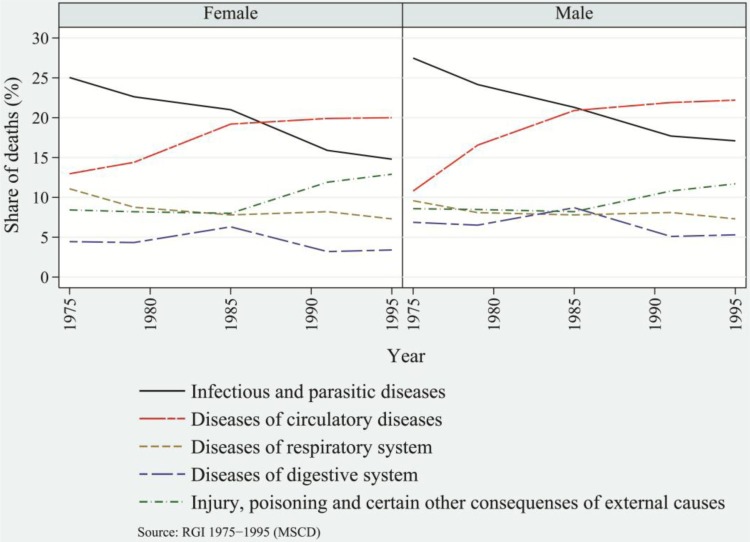
Share of major causes of death among females and males, urban India, 1972–1997: Infectious and parasitic diseases, circulatory diseases, respiratory diseases, diseases of the digestive system, injury, poisoning, and certain other external causes.

Over time, with the modest decrease in the burden of communicable diseases, NCDs emerged as a major cause of death. The mortality burden attributed to NCDs has been increasing over time without replacing the burden attributed to communicable diseases. Among rural populations, the burden of NCDs increased from 12% in 1977 to 13% in 1982 and 28% in 1997 (40). The decade of the 1990s witnessed a major rise in the burden of NCDs. Among urban populations, the share of NCDs increased from 29% in 1975 to 35% in 1985 and 36% in 1995 (41). In general, the burden of NCDs among urban populations as compared to rural populations has been consistently higher. Among urban populations, the major changes in disease patterns were caused by infectious and parasitic diseases, which declined steeply by 10% during 1975–1995, and diseases of the circulatory system, which increased asymptotically to the highest share and engrossed the largest share of 21% of total burden of diseases in 1995 (Figs. 2 and 3).

Diseases of the circulatory system have been the prominent killers in recent decades as compared to infectious and parasitic diseases, which were the top killer diseases until the 1980s. In contrast to urban populations, throughout the period 1972–1997, rural populations’ top killer diseases had been those of the respiratory system, though the rate has been declining over time. Apart from these prominent causes of deaths, injuries, poisoning, and death caused by other external factors have been significantly responsible for changes in disease patterns. Among rural and urban populations, the burden of this category of diseases increased from an average of 4 and 8.5%, respectively, in early1970s to an average of 11 and 12%, respectively, in late 1990s. In later half of 1990s, among both rural and urban populations, males in general were at greater risk of dying than females. In particular, there were more deaths among males due to infectious and parasitic diseases and to diseases of the circulatory system. These have been the prominent killer diseases, and they have largely shaped the disease patterns.

The recent report on causes of death from 2001–2003 provides percentage share of causes of death by major categories of diseases. The results assert that CVDs are the top killer diseases in rural as well as urban areas and more profoundly among urban males. By age groups, for the age groups of 25–69 and 70+, deaths due to CVDs were highest among urban males. For example, deaths due to chronic NCDs were higher by 4 and 2%, among urban males aged 25–69 and 70+, respectively, than among urban females (22). In 2006, studies by Joshi et al. conducted in the eastern and western Godavari regions of Andhra Pradesh demonstrated similar findings. Death data were recorded using a verbal autopsy method from a well-established cause of death surveillance system for a rural area. Joshi et al. affirm chronic NCDs as the leading cause of death in this area, which indicates the rapid progress of epidemiological transition in rural India (30).

**Fig. 4:**
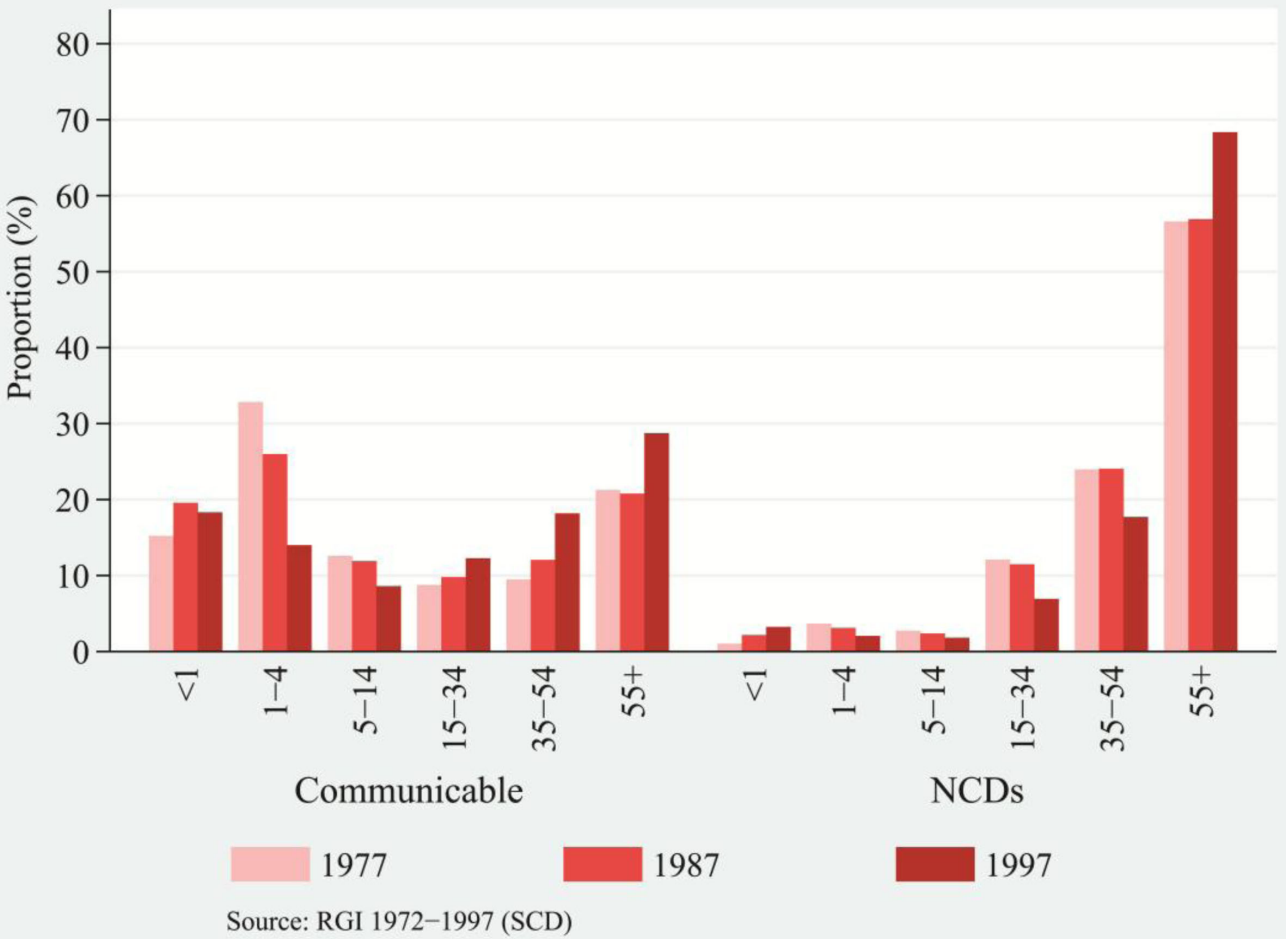
Distribution of deaths attributed to communicable diseases and NCDs, by broad age groups, rural India, 1977, 1987, and 1997.

**Fig. 5:**
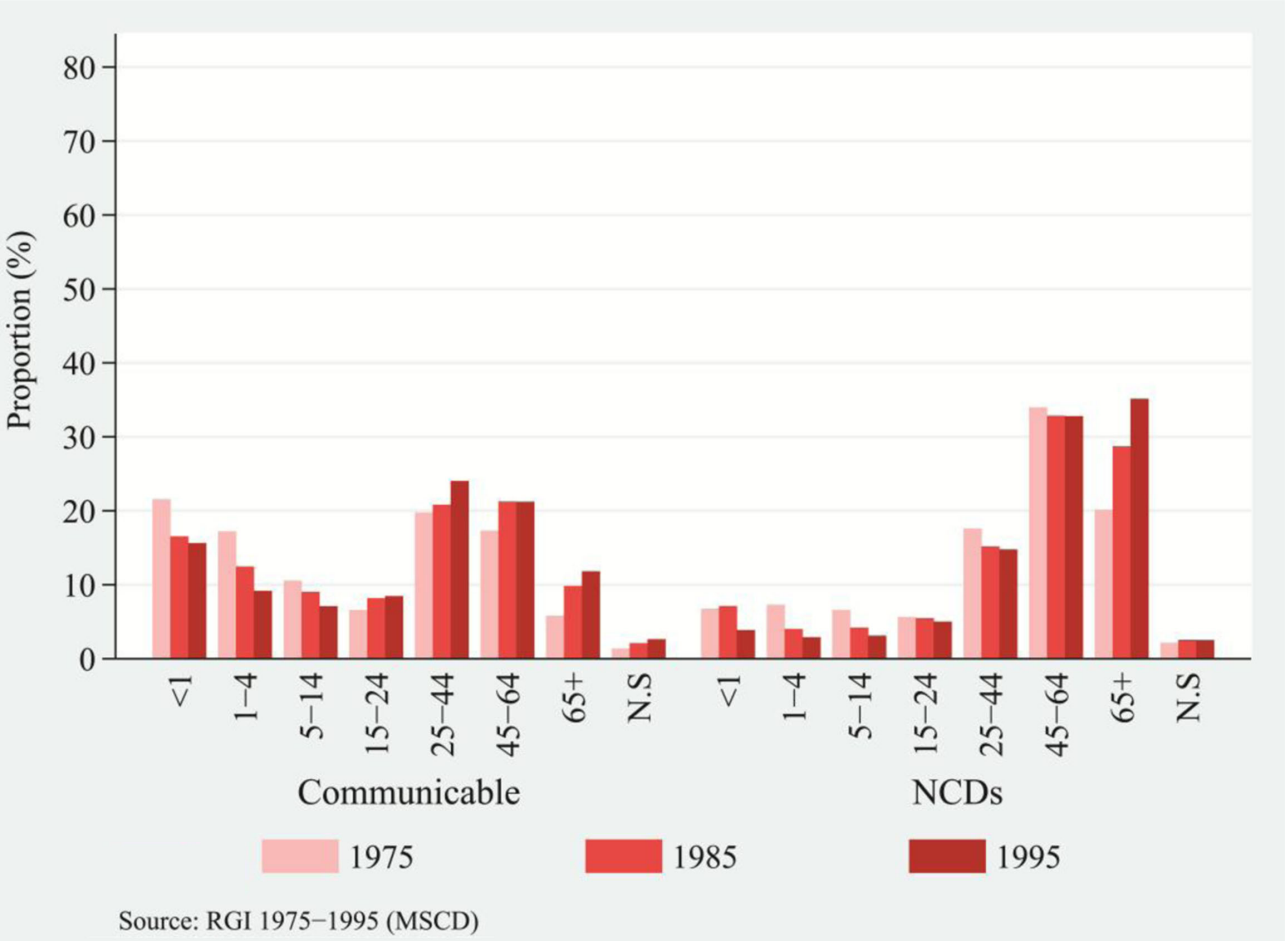
Distribution of deaths attributed to communicable diseases and NCDs, by broad age groups, urban India, 1975, 1985, and 1995.

Figures 4 and 5, for rural and urban population, respectively, show the distribution of deaths attributable to communicable diseases and NCDs by broader age groups separately by residence for selected years (40, 41). For each category of disease by residence, the distribution of death is comparable. Over time, the transformation in the distribution of deaths is apparent. Among rural and urban populations, the burden of deaths due to communicable diseases had been dominant for children aged 0–4. In general, among the adults and the aged population (15 and above age groups), the proportion of deaths due to communicable diseases had been increasing over the time period (Figs. 4 and 5, left panel). The proportion of deaths in adult and old ages was prominent between the mid-1980s and the mid-1990s, indicating faster progress in the shifting of deaths to higher ages from lower ages.

Nevertheless, the transformation in distribution of deaths attributed to communicable diseases seemed modest. Relatively, the transformation in distribution of deaths attributed to NCDs seemed prominent. The shape of distribution of deaths attributed to communicable diseases had been relatively uniform across the ages in comparison to the highly skewed distribution of death attributed to NCDs, depicted in Figs. 4 and 5. Compared with the lower burden of NCDs for children's age groups, older rural and urban populations bore a comparatively higher burden of NCDs. Among rural and urban populations, deaths had been increasingly concentrated in the ages of 55 and above and of 45 and above, respectively (Figs. 4 and 5, right panel). The transformation in distribution of deaths attributed to NCDs unravels a much larger proportion of deaths drifting toward old ages. Comparatively, urban populations experienced a higher burden of NCDs than rural populations over a wide range of ages. There was a rapid increase in the concentration of deaths in older ages from the mid-1980s and the mid-1990s, signaling a rapid transformation in distribution of deaths attributable to NCDs. The fall of mortality rates in adult ages and the resulting increase to 8% in 2011 (54) in the proportion of deaths among the aged population (those that are vulnerable to chronic diseases) demonstrate that the marginal lives in old ages are prone to an elevated risk of dying. Therefore, the age structural transition supplements the transformation in age-at-death and structural changes in disease patterns.

These results confirm that India did not see any fall in the burden of diseases with the progress of demographic and epidemiological transition, but that it moved to a more challenging stage of double burden of disease dominated by burden of NCDs in old ages. However, as may be seen in the progressive stage of epidemiological transition, the rise in the burden of NCDs tends to correspond with the enormous rise in the prevalence rate of chronic diseases. This empirical evidence supports the structural changes in causes of death vis-à-vis transformation in the age pattern of morbidity and mortality. The growing burden of chronic diseases in older ages is a manifestation of structural changes in the disease patterns. These changes in the age patterns of morbidity and in the causes of death structures signal progress in epidemiological transition of India, albeit as a notable variant from the pattern observed for developed countries.

### Mortality transition in India

India experienced a significant rise in life expectancy at birth (e_0_) during the reference period of the study. The e_0_ of India among females and males–increased by 14 and 19 years, respectively, from 1970–75 to 2006–10 (13, 23), signifying marked improvement in the health status of the population. The states of India display more insightful patterns. Among rural females of Kerala, life expectancy reached 77.2 years in 2006–2010, which is no less remarkable progress compared to developed nations. Over time, the IMR and CDR have fallen considerably. In addition, the adult mortality rate fell from 274 to 213 (per 1,000 populations) during 1990–2008 (55). Hence, the population is surviving to higher ages, and as a result, deaths are occurring at higher ages. Aside from this, the growing burden of chronic diseases in older ages and the concomitant structural changes in the disease patterns impel the transformation in the age pattern of mortality.

**Fig. 6:**
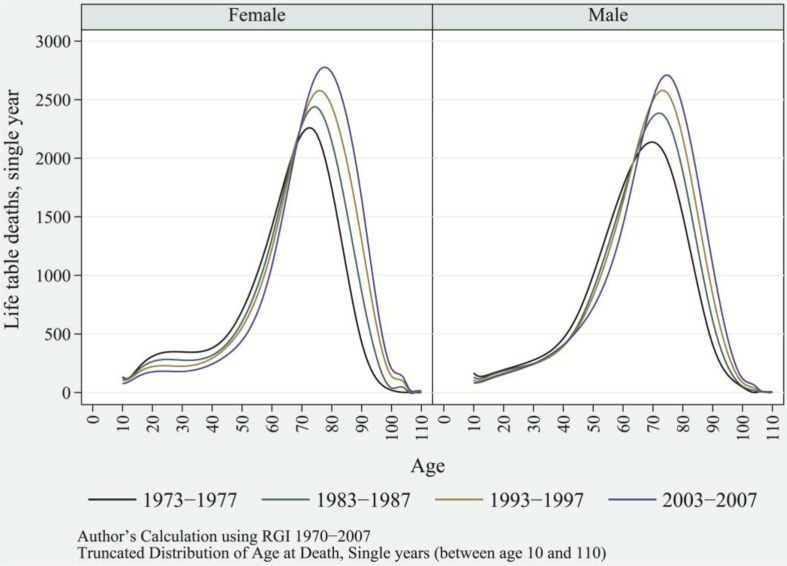
Distribution of age-at-death and modal age-at-death for selected years, female and male, India, 1973–1977, 1983–1987, 1993–1997, and 2003–2007.

Changes in the age pattern of mortality are better understood in terms of distribution of age-at-death. In principle, transformation in distribution of age-at-death complies with the transformation in age pattern of mortality (38). Over time, the fact that deaths are occurring at higher ages is apparent (Fig. 6). Of all the ages, deaths tend to be concentrated near the mode of the distribution of age-at-death, which generally falls in the old ages. As this process progressed, the distribution of age-at-death tended to be unimodal in the 1990s, instead of the bimodal pattern seen in the 1970s and 1980s; this resulted in a definite shape to the distribution of age-at-death. The drifting of deaths toward higher ages and the concomitant concentration of deaths in old ages turned the distribution of age-at-death to a bell-shaped curve in recent decades. This transformation in distribution of age-at-death displays the impressive progression of mortality transition and its ramifications on epidemiological transition.

The progression in mortality transition can be examined by using relevant measures such as the 25th percentile of age-of-death, the 75th percentile of age-at-death, and the modal age-at-death. Table 4 provides the trends of the 25th and 75th percentiles of age-at-death by population categories: rural-urban and female-male at intervals of five years during the period of 1970–2007. Among urban females, the 25th and 75th percentiles of age-at-death increased linearly from age 60 and age 80, respectively, in 1970–1974; and from age 68 and age 85, respectively, in 2003–2007. Among urban males, the 25th and 75th percentiles of age-at-death increased linearly from age 57 and age 78, respectively, in 1970–1974; and from age 62 and age 82, respectively, in 2003–2007. Similar linear increases in percentiles of age-at-death were observed for rural females and males. Though the linearity was similar for all population categories, the 25th and 75th percentiles of age-at-death for urban females remained consistently higher throughout the time period when compared to other categories of population.

**Table 4:** Trends in 25th and 75th percentiles of DOD, by residence and sex, India

	25th percentile of DOD				75th percentile of DOD			
		
Year	Rural female	Rural male	Urban female	Urban male	Rural female	Rural male	Urban female	Urban male
1970–1974	54	54	60	57	76	75	80	78
1974–1978	55	54	60	57	76	75	80	77
1979–1983	58	57	63	59	79	76	81	79
1984–1988	59	57	62	59	79	77	82	78
1989–1993	61	58	65	60	79	78	82	79
1994–1998	62	58	65	61	81	78	84	80
1999–2003	63	58	66	61	82	79	84	82
2003–2007	64	60	68	62	83	80	85	82
Adj. *R* ^2^ *	0.9678	0.8694	0.9362	0.9414	0.9502	0.9451	0.9099	0.8485

Source: Author's calculation from RGI (25); DOD: distribution of age-at-death.

* Adj. *R*^2^ is based on 34 observation of percentile age-at-death between 1970 and 2007.

As supported by the findings of Wilhelm Lexis in 1877 (49), life table deaths move up in the left-hand slope of the distribution of age-at-death, implying a reduction in premature mortality. Among rural and urban females, the 25th percentile of age-at-death shifted to the right by 8 and 10 years, respectively. This shift had been passive among males as compared to females. As life table deaths drifted to higher ages, the left-hand slope of the distribution of age-at-death tends to be more vertical, moving most deaths near the modal age-at-death. Therefore, greater reduction in premature mortality, or alternatively the rapid fall in adult mortality, results in the concentration of deaths in old ages and the overall transformation in age-at-death. Correspondingly, the right-hand slope of the distribution of age-at-death also tends to be vertical; however, this shift is weaker compared to developed nations (Fig. 6) (35).

**Fig. 7:**
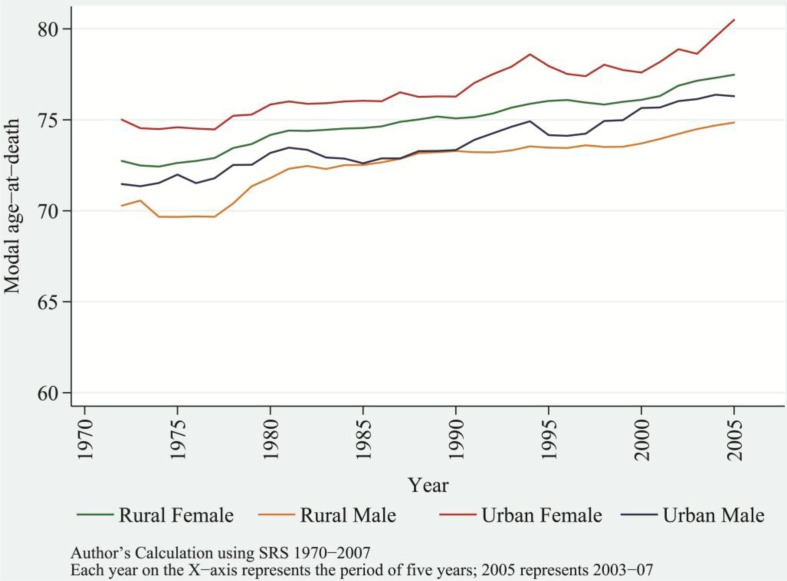
Trends in modal age-at-death by sex and residence, India, from 1970–1974 to 2003–2007; rural female, rural male, urban female, and urban male.

As a result of this process, most deaths are concentrated near the modal age-at-death, leading to rises in the modal values of distribution of age-at-death. With the rise in modal values, the modal age-at-death increased linearly, *r* ^2^=0.9615 for females and *r* ^2^=0.9120 for males. Among urban females and males, modal age-at-death in 1970–1974 increased linearly from age 75 and age 71.5, respectively, to age 80.5 and age 76, respectively, in 2003–2007. Among rural females and males, the modal age-at-death increased from age 73 and age 70, respectively, in 1970–1974, to age 77.5 and age 75, respectively, in 2003–2007 (Fig. 7). Throughout the reference periods, females—irrespective of their residence—showed a consistently higher modal age-at-death than did their male counterparts. This is because of higher *e*_*x*_ among females than males, especially in old ages. The simultaneous process of accumulation of deaths near the modal age-at-death and the shift in modal age-at-death toward higher ages casts a thin shape to the distribution of age-at-death. Therefore, this suggests a decline in variance in age-at-death or the process of compression of mortality over the time period (36, 46, 49).

**Fig. 8:**
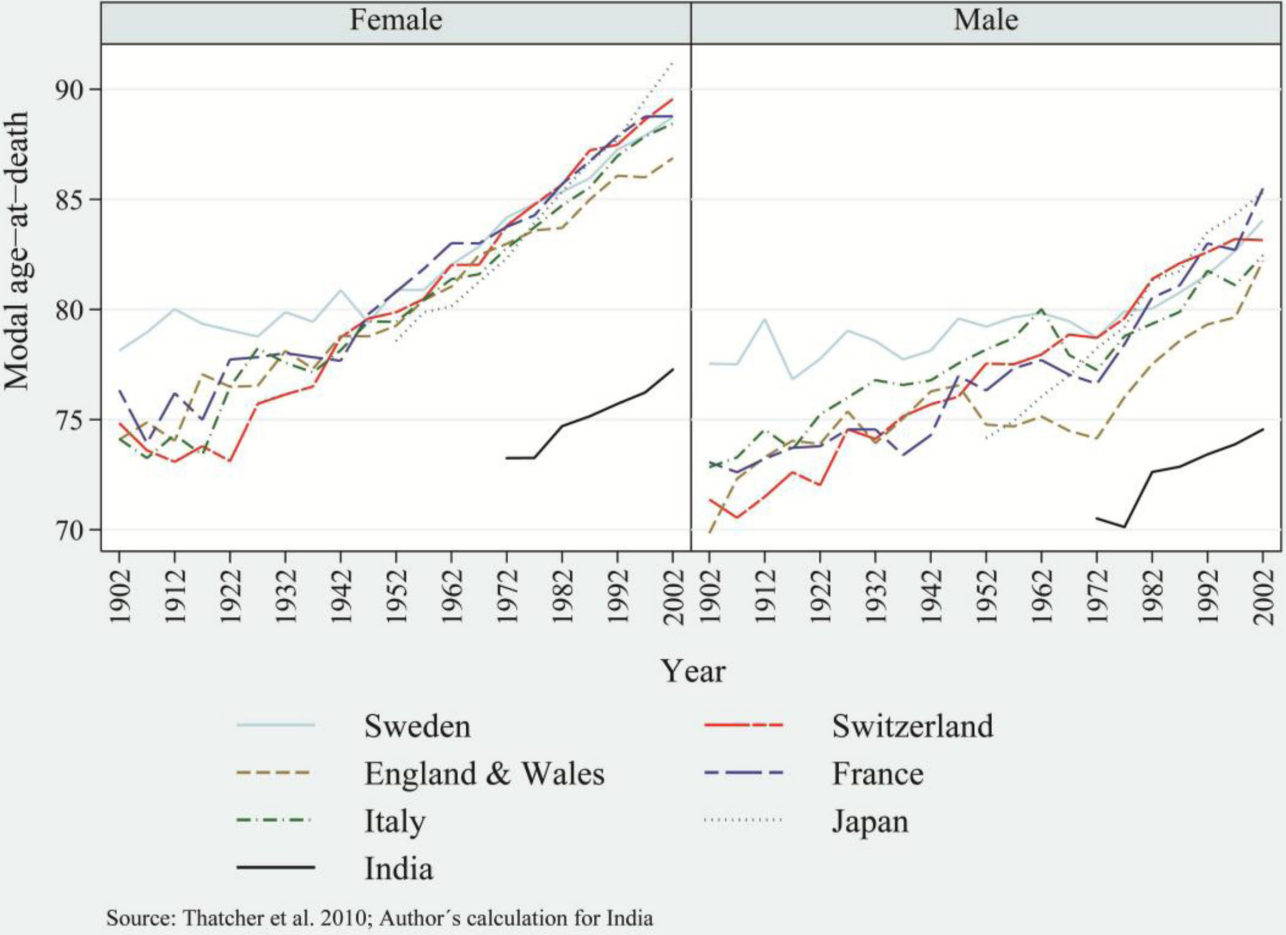
Trends in modal age-at-death among females and males of selected developed countries and India, including Sweden, Switzerland, the UK, France, Italy, and Japan.

Comparisons of modal age-at-death between India and some developed nations (such as Sweden, Switzerland, the UK, France, Italy, and Japan) reveal that India's modal age-at-death in recent decades corresponds to modal age-at-death of developed countries in the 1930s and 1940s (Fig. 8a and 8b) (37). Therefore, India significantly lags behind developed nations in the progress of mortality transition—that is, in demographic and epidemiological transition (28). Nevertheless, in recent decades, a sustained and rapid decrease in mortality rates and the linear increase in *e*_*x*_, the 25th and 75th percentiles, and in the modal age-at-death provide an irreversible and definite shape to the distribution of age-at-death manifested as advances in mortality transition.

The progression in mortality transition is evident and phenomenal. Most older age deaths have been characterized by and attributed to the increasing prevalence of chronic diseases and fatality from them. In addition, the increasing burden of communicable diseases in old ages augments the number of total deaths. Altogether, the advances in mortality transition are concomitant with the structural changes in disease patterns vis-à-vis mounting age patterns of morbidity and mortality. In sum, these phenomena provide evidence of rapid progress in demographic and epidemiological transition.

## Discussion and conclusion

In this study, we examined the structural changes in patterns of diseases vis-à-vis advances in mortality transition, which unraveled the considerable progress of epidemiological transition. From 1970 to 2007, India moved swiftly from the dominance of child and adult mortality to a progressive phase dominated by old age mortality. Although the initial periods of the 1970s and 1980s were characterized by a larger burden of communicable diseases, the burden of chronic NCDs emerged as a major cause of old age deaths in the later period even though communicable diseases responsible for deaths have been interchanged. Currently, the burden of communicable diseases remains substantially higher and is accountable for more than a 30% share of all deaths (15, 22, 32).

Although the burden of communicable diseases impacted a variety of ages during the 1970s and 1980s, by the mid-1990s, the burden of communicable diseases had increased considerably in adult and old ages. At the same time, the burden of chronic NCDs has been increasing in old ages; therefore, those who survived through their early years to middle and old age are susceptible to an elevated risk of dying. The mounting age pattern of chronic diseases in recent decades corroborates the higher death toll attributed to NCDs in old ages, and the emerging age pattern of mortality confirms the process of postponement of death. Among urban aged females, the prevalence rate of hypertension and problem of joints and bones rose from 20.4 and 22.3, respectively, in 1995–1996 to 80 and 32, respectively, in 2004. Among urban males, chronic diseases such as heart disease and diabetes rose from 15.4 and 15.5, respectively, in 1995–1996, to 45 and 57, respectively, in 2004 (Table 1). Hence, in recent decades, the enormous increase in the prevalence rate of chronic diseases and resulting fatalities has been responsible for structural changes in disease patterns.

The results showed remarkable progress in mortality transition consistent with structural changes in disease patterns. The emerging bell shape of the distribution of age-at-death manifests as an increasing bulk of deaths near the modal age-at-death. The phenomenon is being propelled by the linear increase in *e*_*x*_, in the 25th and 75th percentiles of age-at-death, and in the modal age-at-death. The modal age-at-death shifted by almost 5 years compared to an increase of 2.7 years in e_70+_ during 1970–2007. The shift in modal age-at-death evidently demonstrates the dominance of old age mortality over the childhood/adult age mortality (34). Globally, the older age mortality is characterized by the heavy burden of NCDs. However, India has moved to a more challenging stage of demographic and epidemiological transition and has experienced a double burden of diseases. India's evolving stage of epidemiological transition generally is not seen among developed nations, where mortality and morbidity are compressed in later years of life. In India, death is concentrated in later years of life but is accompanied by a mounting burden of morbidity. Comparatively, India's mortality conditions lag behind those of developed nations by almost 60–70 years. Continued progress in mortality transition and structural changes in the disease patterns accompanied by a linear rise in *e*_*x*_ indicates that there is a compelling variation in advances found so far in epidemiological transition witnessed by the developed nations, with similar matrices for India.

Amidst demographic and epidemiological transition, India is experiencing a remarkable transformation in the age pattern of morbidity and mortality, the structural changes in disease patterns and consequent double burden of diseases and in mortality transition. Progression in such fundamental and integral demographic processes is evidence of advances in epidemiological transition in the last four decades. All the categories of population, that is, rural-urban and female-male are advancing in epidemiological transition. Urban females have experienced the greatest structural changes in disease patterns and mortality transition compared to other population categories and are leading in epidemiological transition.

##

**Main findings**

- India is currently experiencing the double burden of communicable and non-communicable diseases. In recent decades, the age pattern ofmorbidity has been rising, primarily due to increased prevalence of chronic diseases, resulting in significant structural changes in disease patterns.
- Modal age-at-death is rising in India, demonstrating the dominance of old age mortality over childhood/adult mortality; this is mostly attributable to the high prevalence of chronic diseases and ensuing fatality.
- Similar to developed nations, most Indians now live to old age; however, they are experiencing increasing number of years lived with disability as a consequence of increasing morbidity. Continued reductions in mortality and structural changes in disease patterns strongly indicate epidemiological transition in India, as these patterns begin to emulate those seen in developed nations.

**Key messages for action**

- India is experiencing rapid health transition, including increased life expectancy at old ages (e_60 and above_). However, the older population is living in poor health. Comprehensive health interventions are required for prevention and control of chronic diseases.
- India is experiencing rapid health transition, including increased life expectancy at old ages (e_60 and above_). However, the older population is living in poor health. Comprehensive health interventions are required for prevention and control of chronic diseases.
- In terms of mortality transition, India lags behind developed nations. The combination of a double burden of disease with high morbidity rates presents challenges for improving the overall health status of the population and necessitates a comprehensive policy and action to prevent and control this burden and promote healthy ageing.
